# Supplementary material for: Clinical characteristics of 199 discharged patients with COVID-19 in Fujian Province: A multicenter retrospective study between January 22nd and February 27th, 2020
Source: PLoS One. 2020 Nov 12;15(11):e0242307. doi: 10.1371/journal.pone.0242307 (PMC7660474; doi:10.1371/journal.pone.0242307)
Supplement: Attachment — Submitted filename: Response to Reviewers.docx [file pone.0242307.s003.docx]

Dear Editors and Reviewers,

Many thanks for your letter and the comments from the reviewers. We are grateful to you and the reviewers for offering us valuable suggestions to help us further improve our work. We have made clarifications and corrections according to your comments. We are grateful for all your efforts and help given to our research. We have studied the reviewers’ comments carefully, and made corresponding revisions as required. The following are the answers and revisions we have made on an item by item basis.

**Comments:**

The manuscript is still suffering from many writing errors. If the authors could have a native English speaker and writer to review the manuscript for flow, subject-noun agreement, and to ensure that everything is written in the past tense, the manuscript would then be more suitable for publication.

**Reply:** Your suggestions really means a lot to us. We feel sorry for our poor writings, and we have invited a native English speaker and writer from USA to help correct and polish our article carefully.

In addition, the authors should make the following changes:

Because you did not do a statistical comparison, please change line 203 to “suggesting that the early outbreak in Fujian may have been milder than that in Wuhan.” (instead of the current wording "indicating..")

**Reply:** Thanks for your kind suggestion. We have changed the sentence in line 203 to “suggesting that the early outbreak in Fujian may have been milder than that in Wuhan.” (Page 16, line 206-207).

Please update the section on corticosteroids in the discusson section to include recently published data in JAMA supporting the use of corticosteroids in severe and critical COVID-19 patients and removing less relevant publications on corticosteroids.

**Reply:** Thanks for your serious suggestion. We have added the newly results published in JAMA on the administration of corticosteroids in critical ill patients with COVID-19 (Page 18, line 252-254) and less relevant publications was removed in the manuscript.

Here we must express our sincere gratitude to you and the reviewers once again, for giving us chances to revise again. All of these comments have contributed a lot to improve the quality of our article. We have invite a native speaker and writer to help polish the manuscript and these changes will not influence the content and framework of the paper. We hope the correction will meet with approval. We really appreciate for your warm work earnestly, and would be glad to respond to any further questions and comments that you may have.

Thank you once again!
